# Bidirectional Mapping of Brain MRI and PET With 3D Reversible GAN for the Diagnosis of Alzheimer’s Disease

**DOI:** 10.3389/fnins.2021.646013

**Published:** 2021-04-09

**Authors:** Wanyun Lin, Weiming Lin, Gang Chen, Hejun Zhang, Qinquan Gao, Yechong Huang, Tong Tong, Min Du, the Alzheimer’s Disease Neuroimaging Initiative

**Affiliations:** AbbVie, Alzheimer’s Association; Alzheimer’s Drug Discovery Foundation; Araclon Biotech; BioClinica, Inc.; Biogen; Bristol-Myers Squibb Company; CereSpir, Inc.; Cogstate; Eisai Inc.; Elan Pharmaceuticals, Inc.; Eli Lilly and Company; EuroImmun; F. Hoffmann-La Roche Ltd and its affiliated company Genentech, Inc.; Fujirebio; GE Healthcare; IXICO Ltd.; Janssen Alzheimer Immunotherapy Research & Development, LLC.; Johnson & Johnson Pharmaceutical Research & Development LLC.; Lumosity; Lundbeck; Merck & Co., Inc.; Meso Scale Diagnostics, LLC.; NeuroRx Research; Neurotrack Technologies; Novartis Pharmaceuticals Corporation; Pfizer Inc.; Piramal Imaging; Servier; Takeda Pharmaceutical Company; and Transition Therapeutics; ^1^College of Physics and Information Engineering, Fuzhou University, Fuzhou, China; ^2^Fujian Provincial Key Laboratory of Medical Instrument and Pharmaceutical Technology, Fuzhou University, Fuzhou, China; ^3^School of Opto-Electronic and Communication Engineering, Xiamen University of Technology, Xiamen, China; ^4^Department of Pathology, Fujian Cancer Hospital & Fujian Medical University Cancer Hospital, Fuzhou, China; ^5^Fujian Provincial Key Laboratory of Translational Cancer Medicine, Fuzhou, China; ^6^Imperial Vision Technology, Fuzhou, China; ^7^School of Data Science, Fudan University, Shanghai, China

**Keywords:** Alzheimer’s disease, multi-modality, image synthesis, 3D CNN, reversible GAN

## Abstract

Combining multi-modality data for brain disease diagnosis such as Alzheimer’s disease (AD) commonly leads to improved performance than those using a single modality. However, it is still challenging to train a multi-modality model since it is difficult in clinical practice to obtain complete data that includes all modality data. Generally speaking, it is difficult to obtain both magnetic resonance images (MRI) and positron emission tomography (PET) images of a single patient. PET is expensive and requires the injection of radioactive substances into the patient’s body, while MR images are cheaper, safer, and more widely used in practice. Discarding samples without PET data is a common method in previous studies, but the reduction in the number of samples will result in a decrease in model performance. To take advantage of multi-modal complementary information, we first adopt the Reversible Generative Adversarial Network (RevGAN) model to reconstruct the missing data. After that, a 3D convolutional neural network (CNN) classification model with multi-modality input was proposed to perform AD diagnosis. We have evaluated our method on the Alzheimer’s Disease Neuroimaging Initiative (ADNI) database, and compared the performance of the proposed method with those using state-of-the-art methods. The experimental results show that the structural and functional information of brain tissue can be mapped well and that the image synthesized by our method is close to the real image. In addition, the use of synthetic data is beneficial for the diagnosis and prediction of Alzheimer’s disease, demonstrating the effectiveness of the proposed framework.

## Introduction

Alzheimer’s disease (AD) is a common neurodegenerative disease and there is no cure for AD so far. Relevant researches show that AD accounts for approximately 60–70% of patients with dementia. Different modalities of neuroimaging can reflect disease changes of AD from different perspectives. Recent studies have shown that MR images and PET images contain complementary information in AD diagnosis (Liu et al., 2017). However, it is difficult to obtain complete modality data for all individuals. Subjects may lack a specific modality due to the high cost and the usage of radioactive tracers, which will increase lifetime cancer risk. In clinical practice, subjects are more willing to accept MRI scans than PET scans due to price and safety considerations. Therefore, collecting a large number of paired data in AD research is a challenge.

In Calhoun and Sui (2016) study, they directly discard samples with incomplete modalities. This reduces the number of samples available for training. The lack of training data may lead to the overfitting problem, thus resulting in poor diagnosis performance. In the past few years, studies on medical image synthesis tasks have been performed. They use algorithms to estimate missing data instead of simply discarding incomplete samples. For example, Li et al. (2014) applied a 3D convolutional neural network (CNN) model to predict PET images from MR images. Moreover, Pan et al. (2018) proposed a 3D-cGAN model to estimate the corresponding PET data based on MRI data. The synthetic data is used for AD classification (Pan et al., 2019) developed a deep learning model called disease-image specific neural network (DSNN), which can simultaneously perform image synthesis and disease classification tasks. Additionally, Nie et al. (2017) synthesized CT images from corresponding MR images using a cascaded 3D full-convolution network (Zhao et al., 2018) adopted Deep-supGAN learning maps between 3D MR data and CT image. Similarly, Bi et al. (2017) synthesized high-resolution PET images from paired CT images. BenTaieb and Hamarneh (2017) attempted to solve the staining problem by training cGAN and task-specific networks (segmentation or classification models) (Wei et al., 2019) predicted PET-derived demyelination from multiparametric MRI. The above studies demonstrate that GAN is a powerful technique for data simulation and expansion in segmentation or classification tasks. However, there is still much room to improve the performance in many medical image synthesis tasks. Some state-of-the-art methods are one-way image synthesis (for example, generating PET from MRI, generating CT from MRI, etc.), which cannot maximize the expansion of missing datasets. Other methods have used very complex preprocessing steps, resulting in high computational costs and difficult in reproducing their results.

In this paper, we imputed the missing data through the 3D Reversible Generative Adversarial Network (RevGAN), and compared the advantages and disadvantages of using synthetic full image and synthetic ROI image. In addition, we also use the synthesized data for AD classification prediction. The main contributions of this study are as follows: First, the reversible structure was utilized to yield better reconstruction ability in the image synthesis experiment. We have improved the generator part, and only one generator was needed to realize bidirectional image synthesis in the proposed method rather than the two generators that were used in the methods of Pan et al. (2018). By adopting the generator together with the stability of reversible architecture, this allows us to train deeper networks using only modest computational resources. Second, by comparing the synthesis experiment of full image and ROI image, we can find that the structural and functional information of brain tissue can be mapped well, but it is difficult to map the structure information such as the skull of the MR image from the PET image. Third, in the classification experiment using full image, we can find that the classification model is mainly based on the brain tissue area in the neuroimaging and is not sensitive to the skull and other structures by comparing the experiment using real MRI and synthetic MRI. Fourth, by comparing the missing data and the use of synthetic data, our proposed image synthesis method is not only of high image quality but also contains disease information about AD, which can be beneficial for the auxiliary diagnosis of diseases. In addition, we also designed a multi-modal 3D CNN model that performed well on the data we used. In the following section, we will first introduce the dataset that we used for evaluation in section “Materials.” We introduce the preprocessing steps and the details of the proposed method in section “Methods.” The experiments and results are shown in section 4 “Experiments and Results,” which mainly includes three parts: experimental setup, image reconstruction and the impact of using synthetic data on Alzheimer’s diagnosis and prediction. The discussion and the conclusion of our work are described in sections “Discussion” and “Conclusion,” respectively.

## Materials

### Datasets

The data used in this study comes from the Alzheimer’s Disease Neuroimaging Initiative (ADNI) database, which publicly provides a series of test subjects’ MRI, PET, other biomarkers and related diagnostic information, providing researchers with A set of standard research data used to study the pathogenesis of Alzheimer’s disease. The data provided by it contains four sets of sub-libraries, namely ADNI-1, ADNI-2, ADNI-3, and ADNI GO. These four stages contain data from subjects in three categories: cognitively unimpaired (labeled as CN), mild cognitive impairment (MCI) and AD. In the problem of predicting the conversion of MCI to AD, it is necessary to review the condition of the MCI subjects so as to keep up with the progress of the subject’s condition. Data will be collected again six months, twelve months, eighteen months, twenty-four months, and thirty-six months after the baseline data is collected. Generally speaking, the time standard for judging whether MCI is converted is thirty-six months. Subjects who converted from MCI to AD within 36 months belonged to developmental mild cognitive impairment (pMCI), and vice versa were classified as stable mild cognitive impairment (sMCI). Although there is no cure for AD so far, the ADNI database has greatly facilitated research on AD by researchers. Table 1 summarizes the details of the data we used.

**TABLE 1:** Summary of the studied subjects and images from the dataset.

Class	Subject Number	Image Number
AD	362	647
CN	308	707
pMCI	183	326
sMCI	233	396

## Methods

**FIGURE 1:**
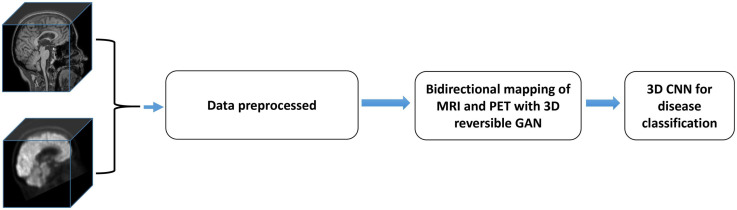
The overall flow chart of the proposed method. It includes three parts, and the data is preprocessed first. RevGAN was then used to synthesize MRI and PET and to evaluate the image quality. Finally, we verify the impact of the synthesized images on disease classification.

There are three major steps in the proposed framework: (i) Data preprocessing step; (ii) Missing data completion using 3D Reversible GAN; (iii) Disease classification using 3D convolutional neural networks. The overall framework of the proposed approach is shown in Figure 1, and the above steps are introduced in the following subsections, respectively.

In our research, we assume M^*k*^ and P^*k*^ are the MRI data and PET data for the kth subject, respectively. The diagnosis result of the model can be expressed as:

(1) yk∧k=F(Mk,Pk),

where yk∧k is the predicted label (such as sMCI/pMCI) for the k^*th*^ subject. If the k^*th*^ subject does not have PET data (missing P^*k*^), our method will predict a virtual Pk∧k with *M*^*k*^ by their underlying relevance. The diagnosis result of the model can be expressed as:

(2) yk∧k=F(Mk,Pk)≈F(Mk,G(Mk))

G and F are both mapping functions. It can be seen from the above formula that there are two major tasks in our framework, (i) learning a mapping function G for completing the missing data, which is described in section “Data Reconstruction Using 3D RevGAN.” (ii) learning a classification model F for AD diagnosis and prediction, which is then introduced in section “Classification With End-to-End CNNs.”

### Data Preprocessing

**FIGURE 2:**
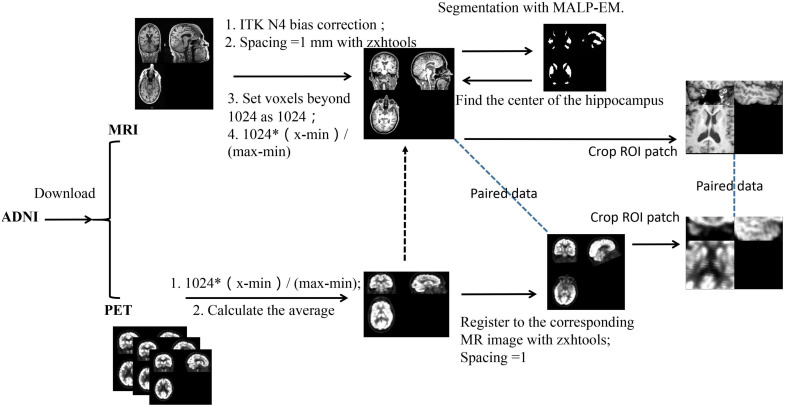
The pipeline of data preprocessing.

All images were preprocessed based on the following steps as described in Huang et al. (2019). The Figure 2 shows the data processing steps. The MR brain images were preprocessed by the standard ADNI pipeline as described in Jack et al. (2008), which includes post-acquisition correction of gradient warping, B1 non-uniformity correction, intensity non-uniformity correction and phantom-based scaling correction. After that, we performed ITK N4 Bias correction on the MR image. Based on zxwtools, MR images were resampled to a 1 mm isotropic world coordinate system and cropped to a size of 221 × 257 × 221. Since the pixel value range of the MR image is long-tailed, we set voxels beyond 1024 as 1024. The pixel values range of the MR images were unified into [0,1024], using the formula of 1024×(input-min)/(max-min). For PET data, each patient may have multiple images, and each Image contains multiple.nii files (30 min to 60 min from taking the medicine, taking pictures at regular intervals). After PET undergoes image quality control (removal of outliers, such as images with 0 mean and variance), we normalize the PET image to [0, 1024] according to the formula 1024×(input-min)/(max-min). After that, we averaged all PET images of the same patient under the same radiography. Finally, PET images were registered to the corresponding MR based on zxhreg through rigid registration. After the registration, PET images were resampled to a 1 mm isotropic spacing.

In this study, the hippocampus region was selected as the ROI. We obtain the center of the ROI as follows: First, for each subject, the hippocampus was segmented using the MALP-EM (Heckemann et al., 2015; Ledig et al., 2015). After getting the segmentation result, we directly calculated the center of the hippocampus (the segmentation result already shows this value). After that, we randomly selected MR data as a template and then registered other MR images to the template through affine registration to obtain a parameter matrix of affine transformation. After obtaining the affine matrix, we map the hippocampus center point on the template to each MR image through the inverse transformation of the affine transformation. Since MR and PET have been registered before, the ROI center of the obtained MR can also be used as the ROI center of PET. After determining the ROI center of the image, we cropped a 96 × 96 × 48 voxel hippocampus region from the image.

**FIGURE 3:**
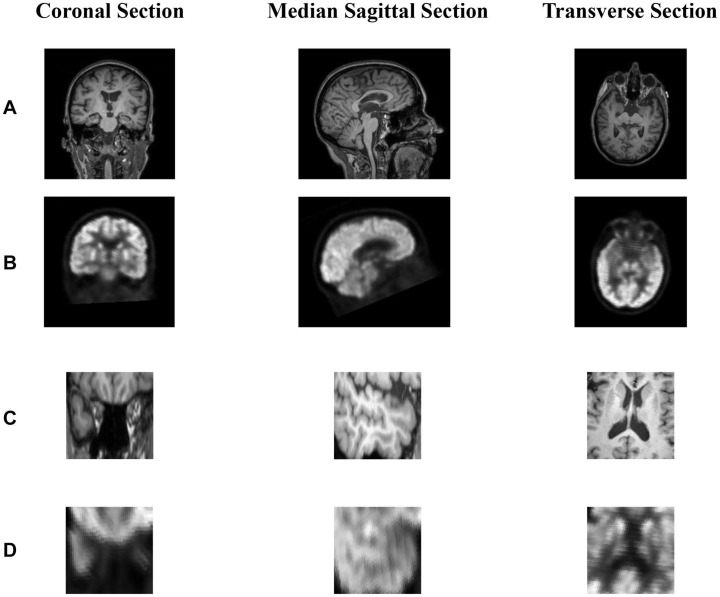
**(A,B)** show the coronal section, median sagittal section and transverse section of MR image and PET image, respectively. **(B)** is registered to the corresponding **(A)** through rigid registration. The dimensions of **(A,B)** are the same as 221 × 257 × 221. **(C)** is the area near the hippocampus of the MR image and **(C)** is obtained from **(A)**. Since **(A,B)** are registered, we can obtain **(D)** corresponding to **(C)** from **(B)**. In short, **(A,B)** or **(C,D)** represents the same area in the brain.

Before the data enters the model, the pixel values range of the data is unified into [0,1] using the formular of (input-min)/(max-min). The full image and the corresponding ROI image are shown in Figure 3.

### Data Reconstruction Using 3D RevGAN

**FIGURE 4:**
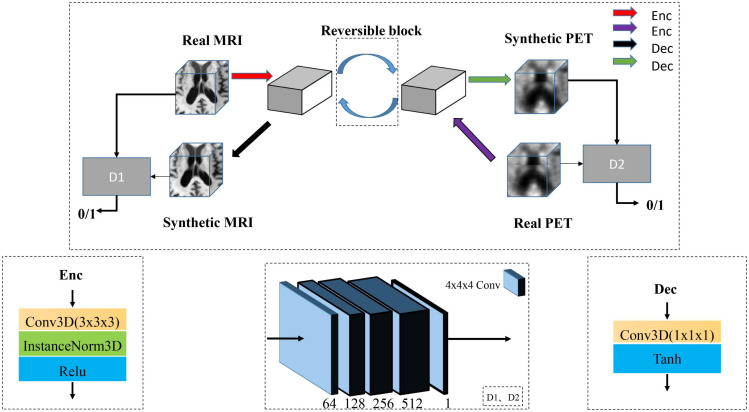
Illustration of our proposed image synthesis method using 3D-RevGAN for learning the mappings between MRI and PET. Encoders Enc (red) and Enc (purple) lift/encode from the image space into the feature spaces. Decoders Dec (black) and Dec (green) project/decode back to the image space. Compared with CycleGAN using two generators, RevGAN only uses one generator to complete the bidirectional mapping from MRI to PET and PET to MRI.

**FIGURE 5:**
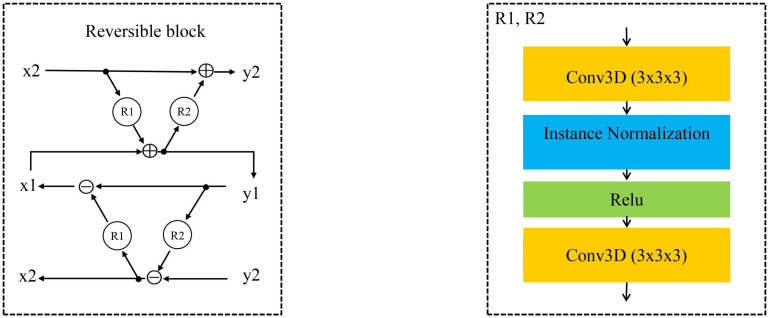
The reversible block is composed of R1 and R2.

In recent years, a lot of studies have widely used GAN (Goodfellow et al., 2014; Yu et al., 2017) in the field of medical image generation. Problems such as data shortage and class imbalance can be solved by GAN (Frid-Adar et al., 2018), and help to understand the data distribution and its potential structure. In this study, we propose an image generation model based on the RevGAN (van der Ouderaa and Worrall, 2019). The proposed framework of our 3D RevGAN model is illustrated in Figure 4, which includes a reversible generator (G), and also two adversarial discriminators (D1, D2). The generator consists of three sequential parts (encoder, invertible core, decoder). The encoder part is constructed by a 3 × 3 × 3 convolutional layer for extracting the knowledge of images. The instance normalization layer and ReLU activation function follow the convolutional layer. The encoder maps the image into a higher dimensionality space. The invertible core C and its inverse C^–1^ are composed of many 3D reversible blocks, which can transfer knowledge from the original domain to the target domain. In our model, we use invertible residual layer, as used in Gomez et al. (2017), using additive coupling (Dinh et al., 2014) as a reversible block. In this work, two reversible blocks were used. Each reversible block is composed of a subnetwork R1 and a subnetwork R2 as shown in Figure 5. R1 and R2 are not reversible. This layer is very flexible and easy to implement. The detailed structures of R1 and R2 are shown in Figure 5, which consist of two convolutional layers, one normalization layer and one non-linear activation layer. The network structure is based on SRCNN (Dong et al., 2014). R1 and R2 in Figure 5 satisfy the following relationship:

(3) $$y_{1}=x_{1}+R_{1}\left(x_{2}\right)x_{1}=y_{1}-R_{1}\left(x_{2}\right),$$

(4) $$y_{2}=x_{2}+R_{2}\left(y_{1}\right)x_{2}=y_{2}-R_{2}\left(y_{1}\right),$$

Since core C and its inverse C^–1^ share parameters, C^–1^ will also be trained when training C, and vice versa. Finally, the decoder part is constructed by a 1 × 1 × 1 convolutional layer for constructing the images in the target domain and is directly followed by a tanh activation function. Decoder projects the image back into the low dimensional image space. For the discriminator, we adopt the PatchGAN architecture as proposed in Zhu et al. (2017). It inputs a pair of real image (such as P^*k*^) and synthetic image [G(M^*k*^)]. We combine the adversarial loss (L_*GAN*_) loss with the cycle consistency loss (L_*cyc*_), so the total loss function used in our model is as follows:

(5) $${\text{L}}_{RevGAN}=L_{GAN}\left(G,D1\right)+L_{GAN}\left(G^{-1},D2\right)+\lambda L_{cyc}\left(G,G^{-1}\right)$$

The weighting factor λ (called lambda) is used to control the weight of the cyclic consistency loss in the total loss.

### Classification With End-to-End CNNs

After the missing data of multi-modality have been completed using the above steps, the diagnosis and prediction of AD are then performed using a classifier. In some previous studies, the hippocampus regions have been chosen as the ROI for the diagnosis of AD (Dickerson et al., 2001; Schuff et al., 2009; Salvatore et al., 2015). The ROI selection is an important step in the proposed method in clinical practice. The hippocampus area is of great significance in the diagnosis of AD, and has been used as a biomarker in diagnostic criteria for Alzheimer’s disease. The hippocampal atrophy is highly related to AD and can be reflected in MR and PET images. The brain atrophy of AD patients affects both the structural change and the metabolic function change at the same time. Lots of previous studies have used the hippocampus area as ROI for the prediction and diagnosis of AD. In contrast, if a full image is used, although more disease information can be used, the information of a large number of irrelevant regions in the full image coexist and may hamper the accuracy of the prediction model. The use of the hippocampus as an ROI is not only essential to improve the diagnostic accuracy, but also can reduce the computational burden and reduce the data redundancy. For a fair comparison, we also performed experiments using full images and use scipy.ndimage.zoom to scale the registered full image size from (221, 257, 221) to (110, 128, 110).

CNN has been well applied in the classification and prediction of brain disease. Briefly, CNN extracts feature through convolutional layers and reduces the network parameters through weight sharing and pooling of convolution. Finally, the classification task is completed through the fully connected layer. The subsequent development of many advanced networks such as AlexNet (Krizhevsky et al., 2012), VGG (Simonyan and Zisserman, 2014), ResNet (He et al., 2016) and so on. In our experiment, in order to evaluate the performance of our proposed 3D CNN, we chose the classic model of VGG and ResNet as the baseline model for comparisons.

Although MR and PET share most information of AD disease changes, it should be mentioned that these two modalities also have complementary information (Fan et al., 2008; Wee et al., 2014; Calhoun and Sui, 2016), which can be beneficial for further improvement of AD diagnosis. The process of synthesized PET can generate complementary information. Although this information comes from MRI, the classification model cannot use this information directly. Through our synthesis method, these hidden information are displayed, so that the classification network can obtain more information. For example, elements (5, 6, 7) are in set A, (15, 16, 17) are in set B, and B = A +10. As long as we know the relationship between AB, we can infer each other. However, in our case A does not contain B, and B does not contain A. In this study, the relationship between MRI and PET is a more complex non-linear complementary relationship, and our model is needed to fit the relationship between them. The synthetic network is trained through a large number of pairs of other people’s MRI and PET relationships. During the training process, the synthetic network learns the method of converting the information in the MRI into the corresponding PET information.

**FIGURE 6:**
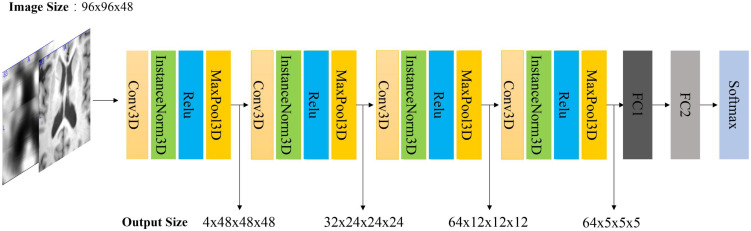
Architecture of our 3D CNN model. Note that if the input is MRI and PET, the input size is 2 × 96 × 96 × 48. The first dimension is the number of channels. FC is a full connected layer. The parameters of FC1 and FC2 are 512 and 2, respectively.

We proposed a 3D CNN model based on ROI crop to learn a classifier and to fuse different features from both MRI and PET. It is worth noting that the general classification model uses batch norm, while we use instance norm (Ulyanov et al., 2016). The output of each convolutional layer is down-sampled by the max-pooling operations. In the 1st, 2nd, 3rd, and 4th layers, the size of the convolutional kernel is set to 1 × 1 × 1, 5 × 5 × 5, 9 × 9 × 9, 5 × 5 × 5, respectively. The number of channels is 4 for the 1st layer, 32 for the 2nd layer, 64 for the 3rd and 4th layers. The classifier consists of 2 FC layers and a softmax layer. After passing the softmax layer, the model outputs the diagnostic label. The detailed model structure is shown in Figure 6.

## Experiments and Results

We have evaluated the effectiveness of our proposed method in the following experiments. Firstly, we evaluated the quality of the synthetic images generated by 3D reversible GAN. Some image quality metrics were used for the evaluation of the reconstructed images. After that, we compared our classification model with other methods including the VGG and ResNet model in the diagnosis task of AD vs. CN. Finally, we verified the impact of the generated PET data in the classification task of Alzheimer’s diagnosis and prediction. Bold values mean the best value in the comparative experiment.

### Experiment Setting

Experimental environment: PyTorch framework, Nvidia GTX1080 GPU. During the image reconstruction training, the Adam optimizer (Kingma and Ba, 2014) was used. The reversible blocks were implemented using a modified version of MemCNN (van de Leemput et al., 2018). Peak signal-to-noise ratio (PSNR), and structural similarity index measure (SSIM) (Hore and Ziou, 2010) are used as evaluation metrics of image quality. RMSE, PSNR and SSIM are the most common and widely used objective image evaluation metrics in image reconstruction. RMSE and PSNR measure absolute errors between source and synthetic images, and SSIM measures structural similarities between them. In the classification experiment, we continue to use Adam as the optimizer. The dataset was separated into training part, validation part and testing part. The ratio of training set, validation set, and test set is 7:2:1. ACC, SEN, SPE, AUC are used as evaluation metrics. These four metrics represent accuracy, sensitivity, specificity and area under the curve, respectively. The higher values of these metrics indicate better classification performance. A 10-fold cross-validation approach was employed to measure and to compare the performance of different methods.

### Results of Image Reconstruction

#### Paired ROI Image

**FIGURE 7:**
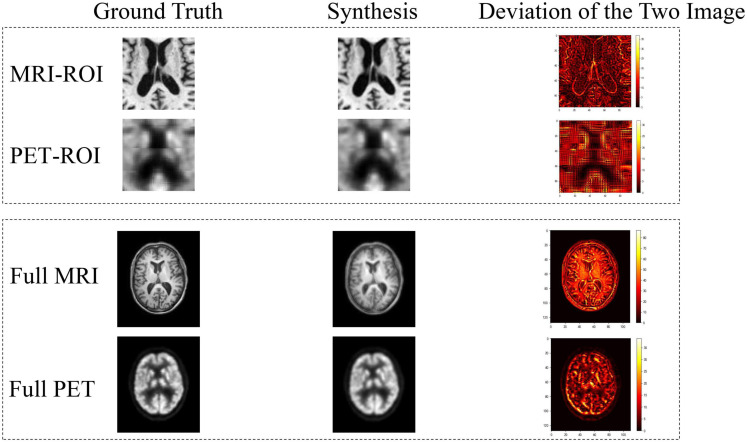
Deviation between real image and synthetic images. In the deviation image, the yellower the color, the greater the error, and the red and black areas represent smaller deviation.

From Figure 7, we can see that our synthetic PET images are very similar to their corresponding real images. When calculating the deviation image, we use matplotlib to draw the heat map, and the deviation image is normalized to [0, 255] for display. There are only positive values in the deviation image, and different slices have different ranges, the largest range is [0, 255]. These results indicate that our trained RevGAN model can generate synthetic PET scans with good image quality that are similar to real PET scans. In addition, we have listed the experimental results in some related papers using different methods in recent years for reference. The results are in Table 2, which shows that the results of different methods are similar in terms of PSNR in PET image reconstruction. It can be seen from Table 2 that our method SSIM reached 0.9389 in MRI image reconstruction, achieving the best performance.

**TABLE 2:** Comparison with others.

		Synthetic PET		Synthetic MRI	
		PSNR	SSIM	PSNR	SSIM
Pan et al., 2020	HGAN	**30.24**	0.6945	26.07	0.6683
Pan et al., 2019	FGAN	29.62	0.6817	25.10	0.6404
Hu et al., 2019	Adversarial U-Net (2D slice)	25.13	−	−	−
Pan et al., 2018	3D-cGAN	24.49	−	−	−
The proposed method (ROI)	RevGAN	29.34	0.8034	**29.81**	**0.9389**
The proposed method (Full Data)	RevGAN	29.42	**0.8176**	24.97	0.6746

*The quality of the reconstructed image. Results of image synthesis achieved by different methods. Just as a reference, because the selected data and data preprocessing are different. Bold values mean the best value in the comparative experiment.*

#### Full Image

To compare the performance based on ROI and that based full images, we have performed a further experiment using full images. The registered full image data (221, 257, 221) is scaled using scipy.ndimage.zoom, and the size is resized to (110, 128, 110), and the resolution of the image is reduced. It can be seen from the results of Figure 7 and Table 2 that the metric of the generated MR image has decreased significantly. Comparing to the use of ROI, the full MR image contains not only the structural information of the brain tissue but also the structural information such as the skull. Although PET images also have complete head coverage, the signal levels of non-brain tissues are quite different compared with brain tissues, so it creates challenges in alignment between the MR images and the PET image. This will lead to decrease the reconstruction performance of image synthesis.

### Results of Disease Diagnosis Prediction

#### Evaluation of our Proposed 3D CNN Classification Model

We chose 3D-VGG11 and 3D-ResNet10 as the baseline models for comparison. We used the same settings for all models to make fair comparisons. The experimental results are shown in Tables 3, 4. The experiment in Table 3 used MRI-ROI data, while the experiment in Table 4 used PET-ROI data. As shown in Table 3, we can see that the ACC and SEN of our proposed 3D CNN model achieved 82.00 and 82.29%, respectively. The experimental results are better than the VGG11 and ResNet10 models. The 3D VGG11 model is slightly better than our model in SPE and AUC metrics. As shown in Table 4, it can be seen that VGG11 has a very good performance on PET images. The ACC, SEN, and AUC reached 89.11, 90.24, and 92.64%, respectively. It is worth noting that our proposed model is higher than the other two models in the SPE metric. If the age-correction processing of Lin et al. (2018) was performed, the performance of the proposed method was improved. The experimental results show that our four-layer model has better performance than the VGG11 and ResNet10 models which have many layers with much more parameters, indicating the effectiveness of our proposed classification method in the diagnosis of AD.

**TABLE 3:** Results (%) of the models trained from only MRI data for CN vs. AD task.

Model	ACC	SEN	SPE	AUC
3D-VGG11	81.19	79.27	**83.45**	**83.67**
3D-ResNet10	80.87	79.63	82.23	81.21
3D CNN (4 layers)	**82.00**	**82.29**	81.69	81.76

*Bold values mean the best value in the comparative experiment.*

**TABLE 4:** Results (%) of the models trained from only PET data for CN vs. AD task.

Model	ACC	SEN	SPE	AUC
3D-VGG11 (Huang et al., 2019)	**89.11**	**90.24**	87.77	**92.69**
3D-ResNet10	86.26	86.56	85.94	84.48
3D CNN (4 layers)	88.77	89.11	**88.41**	87.11

*Bold values mean the best value in the comparative experiment.*

#### Results of Diagnosis Using Synthetic Data

The model used in all multi-modal experiment is our 3D CNN model. We simulated the absence of data and tested the impact of the generated PET on the diagnosis and prediction of AD (Tong et al., 2017) used non-linear graph fusion to fuse the features of different modalities. To take advantage of information about the disease, we adopted a framework to integrate multi-modality information based on our proposed 3D CNN model. In this part of the experiment, MR and PET images are used as two parallel channels. After then, the paired MR image and PET image are stacked as a 4D image. We also employed the above four metrics for performance evaluation of AD diagnosis. Disease classification results were reported in Tables 5–8.

**TABLE 5:** Diagnosis results (%).

	ACC	SEN	SPE	AUC
MRI+PET (Real data)	**89.26**	82.69	**96.48**	**90.98**
MRI+PET (Miss 50% data)	84.64	85.82	83.45	83.92
MRI+50%PET+50% synthetic PET	87.63	87.07	88.24	87.07
MRI+ the other 50% synthetic PET	87.99	87.76	88.24	87.36
MRI+100%synthetic PET	89.05	**90.48**	87.50	87.92

*In this AD vs. CN classification experiment, all MR images are real, but PET images were divided into five cases. The data used in the experiment is an ROI image. “MRI+ the other 50% synthetic PET” is to replace the original real data in “MRI+50%PET+50% synthetic PET” with synthetic data and change the synthetic data to real data. Bold values mean the best value in the comparative experiment.*

**TABLE 6:** Diagnosis results (%).

	ACC	SEN	SPE	AUC
MRI+PET (Real data)	**72.84**	**88.89**	52.78	68.30
PET+MRI (Miss 50% data)	66.87	67.05	66.67	68.95
MRI+50%PET+50% synthetic PET	69.28	55.68	**84.62**	70.29
MRI+ the other 50% synthetic PET	68.49	84.62	50.00	67.53
MRI+100%synthetic PET	71.23	74.36	67.65	**73.66**

*In this pMCI vs. sMCI classification experiment, all MR images are real, but PET images were divided into five cases. The data used in the experiment is an ROI image. Bold values mean the best value in the comparative experiment.*

**TABLE 7:** In this AD vs. CN classification experiment, all PET images are real, but MR images were divided into five cases.

	ACC	SEN	SPE	AUC
MRI+PET (Real data)	**89.26**	82.69	**96.48**	**90.98**
MRI+PET (Miss 50% data)	84.64	85.82	83.45	83.92
PET+50%MRI+50% synthetic MRI	87.12	81.68	92.48	85.59
PET+ the other 50% synthetic MRI	86.71	87.73	85.51	84.92
PET+100%synthetic MRI	88.64	**91.60**	85.71	87.36

*The data used in the experiment is an ROI image. Bold values mean the best value in the comparative experiment.*

**TABLE 8:** In this sMCI vs. pMCI classification experiment, all PET images are real, but MR images were divided into five cases.

	ACC	SEN	SPE	AUC
MRI+PET (Real data)	**72.84**	**88.89**	52.78	68.30
PET+MRI (Miss 50% data)	66.87	67.05	66.67	68.95
PET+50%MRI+50% synthetic MRI	67.81	83.33	50.00	67.87
PET+ the other 50% synthetic MRI	68.67	67.05	70.51	69.81
PET+100% synthetic MRI	71.18	69.07	**73.97**	**70.80**

*The data used in the experiment is an ROI image. Bold values mean the best value in the comparative experiment.*

**FIGURE 8:**
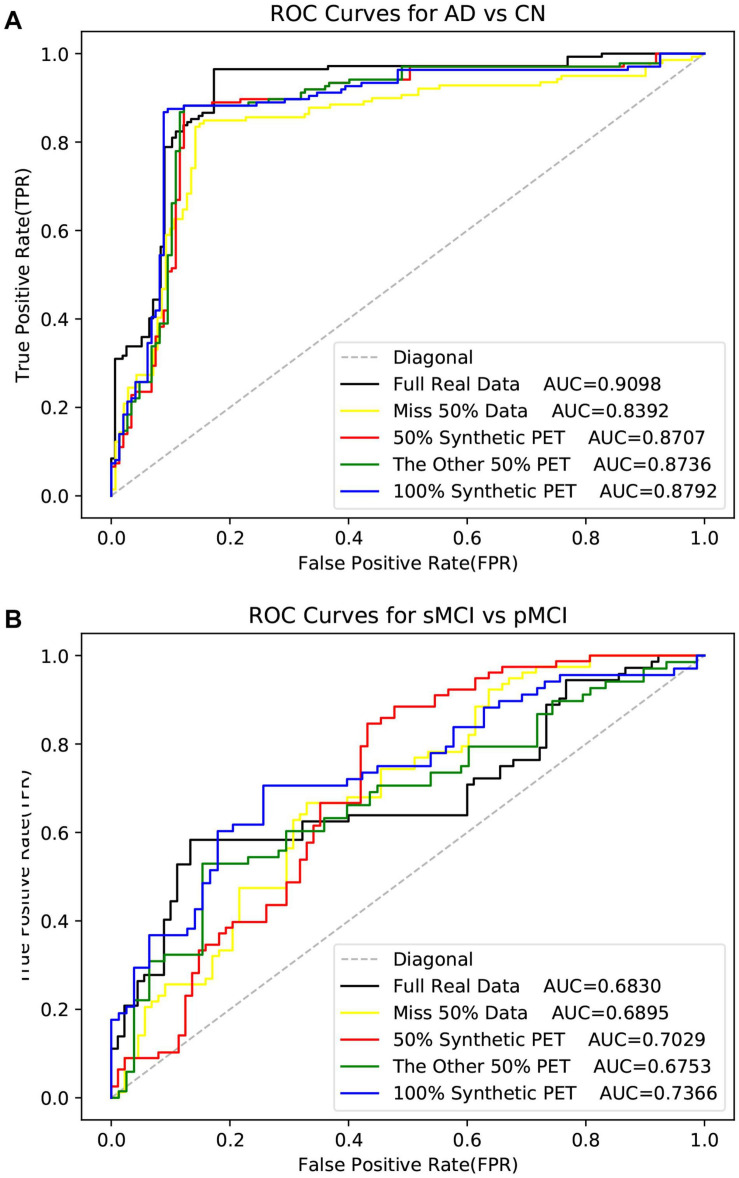
The ROI data is used here. The ROC curves in the experiment of disease classification with the synthetic PET image.

**FIGURE 9:**
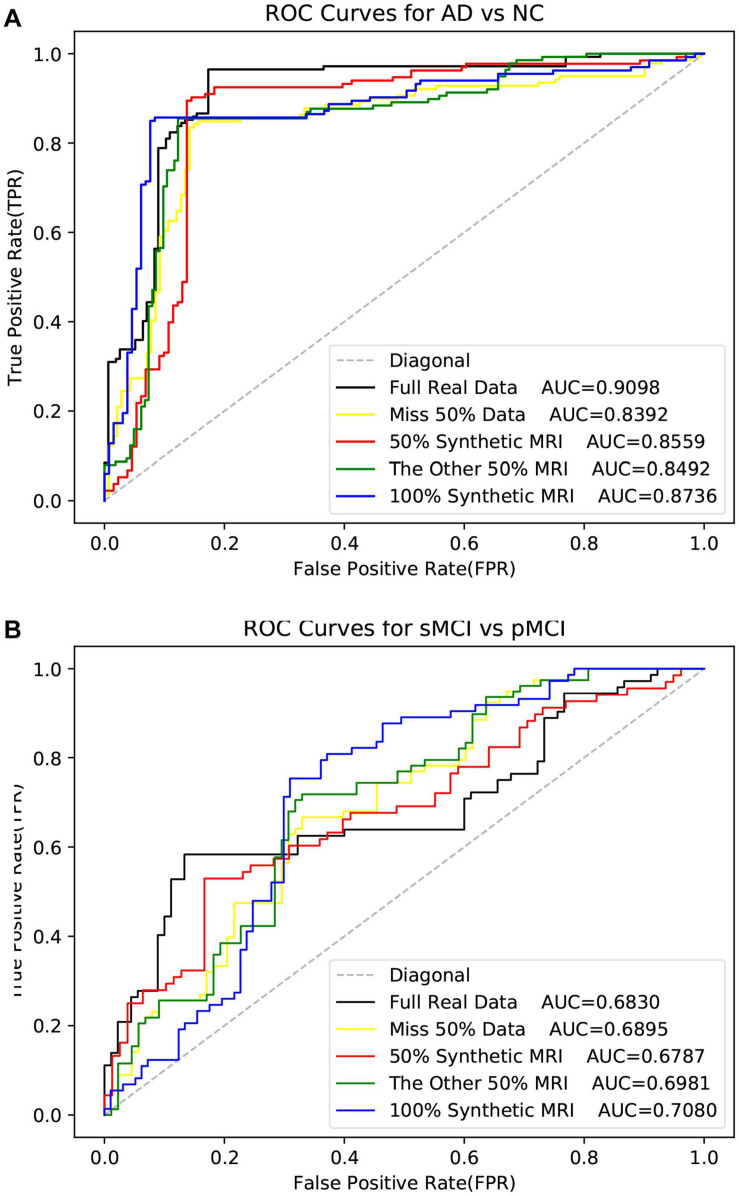
The ROC curves in the experiment of disease classification with the synthetic MR image. The ROI data is used here.

**FIGURE 10:**
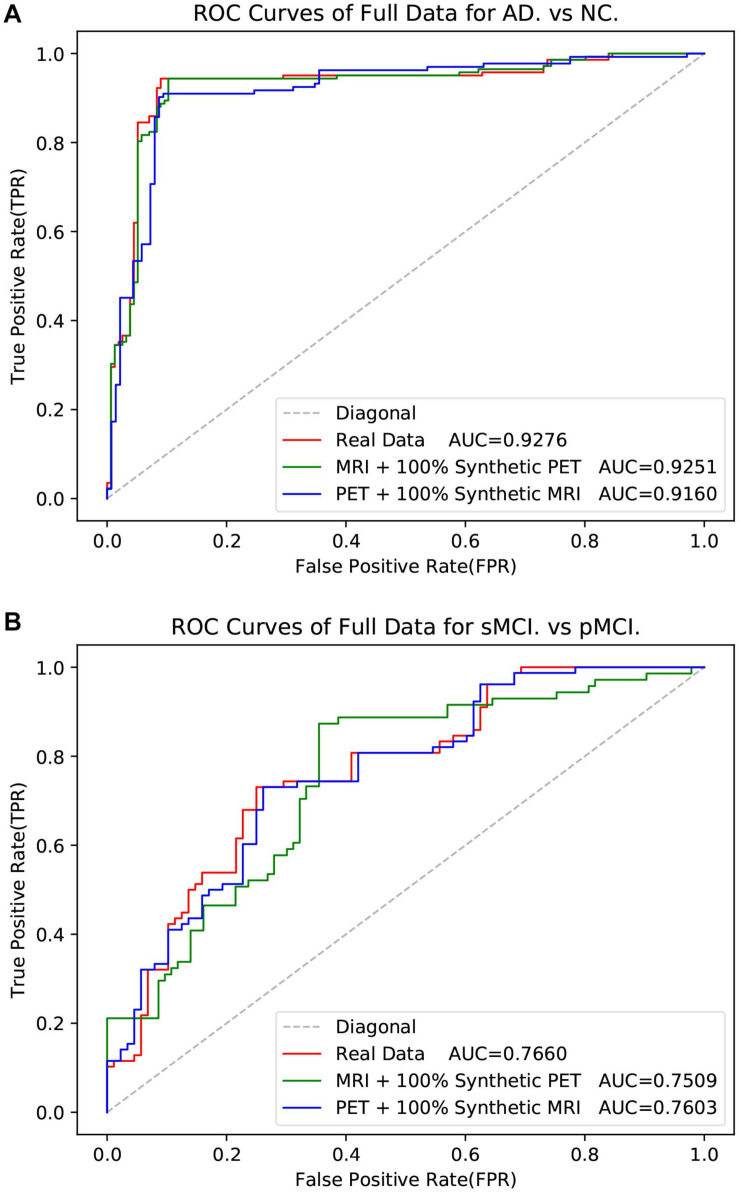
The ROC curves in the experiment of classification with the full data.

As shown in Table 5, the method used real data got the best metrics in terms of ACC, SPE, and AUC. Note that “MRI + 100% synthetic PET” has the highest value in SEN. A similar situation can be seen in Tables 6–8. The method used real data got the best metrics in terms of ACC, SEN, while using 100% synthetic PET has achieved the highest AUC in Table 6. In Table 7, the method using real data obtained the best indicators in terms of ACC, SPE, and AUC. Please note that “PET + 100% synthetic MRI” has the highest SEN value. In Table 8, the method used real data got the best metrics in terms of ACC, SEN, while using 100% synthetic MRI has achieved the highest SPE and AUC. As shown in Tables 5–8, after using synthetic data, there is a higher classification score. This shows that our method is effective. The experimental ROC curves are shown in Figures 8, 9. Moreover, the relevant experimental results using full image are shown in Tables 9, 10, and the ROC curve is shown in Figure 10. In this study, for the classification tasks using full image, resampled images at a lower resolution were used instead of using multiple patches as (Pan et al., 2018). We think that not all patches within the whole image were affected by the disease changes of AD. Some patches from AD subjected may be not affected by AD and their diagnostic label may be fuzzy. Therefore, if the selected patches are not accurate, it will lead to poor performance. In addition, compared with using a full image, using multiple patches will lose the spatial position information between different brain regions.

**TABLE 9:** The AD vs. CN classification experiment uses synthetic full data and real full data.

	ACC	SEN	SPE	AUC
MRI+PET	**92.28**	90.38	**94.37**	**92.76**
MRI+100% synthetic PET	91.95	89.74	94.37	92.51
PET+100% synthetic MRI	90.77	**90.58**	90.98	91.60

*Bold values mean the best value in the comparative experiment.*

**TABLE 10:** The sMCI vs. pMCI classification experiment uses synthetic full data and real full data.

	ACC	SEN	SPE	AUC
MRI+PET	**74.10**	**75.00**	73.08	**76.60**
MRI+100% synthetic PET	73.78	64.52	**85.92**	75.09
PET+100% synthetic MRI	73.49	73.86	73.08	76.03

*Bold values mean the best value in the comparative experiment.*

## Discussion

In the classification task, we propose a 3D CNN model with only four convolutional layers to achieve T1-MRI and FDG-PET AD diagnosis. The hippocampal area was used as ROI for input as it is the most frequently studied and is thought to be of the highest related region of AD. Through our experiments, we found that the proposed four-layer network has a lightweight structure but with competitive performance in AD classification tasks. Especially, we use small-sized kernels in the first convolutional layer to prevent early spatial downsampling in contrast to most standard architectures for image classification. For example, ResNet uses relatively large kernel sizes and strides in the first layer, which dramatically reduces the spatial dimension of their inputs. This can accelerate the computation. However, the loss of a large amount of information at the beginning layer may adversely affect the results, especially in medical images (Liu et al., 2020). In addition, the input size is only 96 × 96 × 48 due to the use of ROI-based data in our experiments. Therefore, premature downsampling can cause severe performance degradation. It can be seen from Table 5 that the performance of “50% synthetic + 50% real” is worse than “100% real” and “100 synthetic.” This indicates that the complexity of the data will affect the performance, because the distribution of synthetic data and actual data may be slightly different, and mixing the two types will make it difficult to train the model. It can be seen from Table 4 that very good performance can be achieved using only PET data. From the Table 6, it is strange that best SPE was achieved with “MRI+50%PET+50%synthetic PET,” and best AUC is achieved with imputed PET (better than when full real data are available). In lots of previous studies, the use of single-modal PET has been able to achieve very good results in the classification of AD. In this work, all multi-modal experiments use the four-layer 3D CNN model we designed, and the best result of single-modal PET is to use the 3D VGG11 of Huang et al. (2019). The models used are inconsistent (Tables 4, 5). These four indicators describe the performance of the model from different aspects, rather than absolute positive correlation. Therefore, due to different experimental settings, some evaluation metrics will fluctuate. In the comparative experiments using real data and synthetic data, the number of data samples is small. AD, NC, pMCI, and sMCI are 647, 707, 326, and 396, respectively. The numbers of samples in different categories are imbalanced. This makes the model biased toward a certain type of sample in order to reduce its own loss, which causes fluctuations in some metrics. When data is missing or synthetic data is used, this situation is more likely to happen.

From Tables 9, 10, it can be seen that although the image resolution becomes lower when using the full image, it still achieves a good classification score. The analysis in “Full Image” shows that the structural and functional information of brain tissue can be mapped very well, but it is difficult to map the structural information such as the skull of the MR image from the PET image. Therefore, it does not perform well when synthesizing a full MRI image from a PET image (Table 2). This problem can be avoided if only the hippocampus ROI is used. Although the quality of the synthesized MR image is not high, the classification result of using the synthesized MR image is not bad (Tables 9, 10). This shows that the model mainly focuses on changes in brain tissue structure (such as hippocampus atrophy, etc.) when diagnosing and predicting diseases, and the structure of irrelevant areas such as the skull has little effect on the results.

In the image synthesis task, reversible blocks are very memory-efficient. Therefore, by adopting the generator together with the stability of reversible architecture, this allows us to train deeper networks using only modest computational resources. Increasing the depth of the reversible block improves the non-linear fitting ability of the model, which can generate higher quality images. Moreover, reversible architecture has been demonstrated to yield superior results when trained using fewer training data in the work of Chang et al. (2018). It can be seen from Table 2 that compared with our work, Pan et al. (2018, 2019, 2020) performed more preprocessing steps to obtain better alignment between MR image and PET images, thus resulting in a good reconstruction results. Despite their advantage on the alignment, our proposed method are still superior to Pan et al. (2018) work on the reconstruction quality of synthetic images in some metrics as shown in Table 2. It is worth noting that too much preprocessing may lose more original information and affect the reliability of the experiment. When registering the PET images to the corresponding MRI, we use nearest neighbor interpolation (Olivier and Hanqiang, 2012). This leads to moiré pattern (Oster and Nishijima, 1963) on the PET image. Compared with the real image, the synthesized PET image has reduced moiré pattern (see Figure 7). Related experimental results are shown in Table 2. From Tables 5–8, we can see that the performance of the method using our synthetic data is superior to those using missing data. The experimental results show that our synthetic PET is beneficial for disease diagnosis. If our synthetic data does not contain disease information, it may hamper the performance of AD diagnosis, leading to worse performance.

The main limitations of this study and future work are as follows. In this study, since the hippocampus is an appropriate ROI selection for the AD diagnosis, we can use the ROI block that removes the redundant information in the data to diagnose and predict the disease and improve performance. When applied to other diseases without a specific target ROI, it is difficult to take advantage of the ROI-based method. Moreover, it would be interesting to show the biologically meaningful measurements of the synthetic data over the real data. In this work, the synthetic PET data were used for AD diagnosis and focused more on the disease changes of AD, rather than the real FDG metabolic changes. The aim of the reconstruction is to improve the classification of AD in the scenario of missing data. The detailed investigation of reconstructing biologically meaningful synthetic data will be carried out in future. Last but not the least, in Tables 5, 6, the ratio was set to 50% (half synthetic PET data) and 100% (all synthetic PET data) in our experiments. By adding more synthetic PET data, the accuracy has been improved by comparing the new results in Tables 5, 6. The effect of the ratio parameter in the random selection of the synthetic PET data will be carefully studied in future work. Finally, the synthesized PET images may be helpful for improving the AD diagnosis performance, while they do not reflect the real metabolic changes of the FDG PET imaging. In Table 3, we have compared the performance of three different CNN models on the AD vs. CN task based on MRI data. Comparing Tables 3, 5, we can find that the performance of MRI+synthetic PET and MRI+PET (Real data) is superior than that of MRI alone due to the complementary information. But, we also believe that there is not a strong relationship between MR and PET in all scenarios, and a good mapping between MRI and PET needs to meet some conditions. If the synthetic model that we trained in the AD diagnosis scene is directly applied to other diseases, it may not work. For example, if we want to apply our method in the scene of tumor diagnosis, a large number of paired MR and PET must be used to retrain the model so that the model can find the relationship between MR and PET in the scene of tumor diagnosis. In this study, hippocampal atrophy in AD patients will be reflected in the structure and functional metabolism of the hippocampus. However, there are many other diseases that may not affect tissue structure and tissue function metabolism at the same time, so it is difficult to find the relationship between MRI and PET. To sum up, whether the disease can cause changes in tissue structure and metabolic function simultaneously may be a condition for MR and PET to be able to map well. Therefore, further research needs to be explored.

## Conclusion

To conclude, we proposed a 3D reversible GAN for imputing those missing data to address the issue of missing data. Specifically, we have also presented a novel 3D CNN architecture to perform classification for AD diagnosis. Moreover, we tested the impact of the synthetic data in the classification task of AD by simulating missing data. During the experiment to evaluate the impact of synthetic data, the multi-modal fusion method by channel fusion (MR images and PET images were stacked into 4D images) is selected. Experiments on the ADNI dataset demonstrate that our method generates reasonable neuroimages. Through the experimental results, we can also find the following three conclusions: First, we can find that the structural and functional information of brain tissue can be mapped well, but it is difficult to map the structure information such as the skull of the MR image from the PET image. Second, the classification model is mainly based on the brain tissue area in the neuroimaging and is not sensitive to the skull and other structures. Third, when the data is missing, the performance of AD diagnosis and MCI conversion prediction can be significantly improved using our method.

## The Alzheimer’s Disease Neuroimaging Initiative

The Alzheimer’s Disease Neuroimaging Initiative is funded by the National Institute on Aging, the National Institute of Biomedical Imaging and Bioengineering, and through generous contributions from the following: AbbVie, Alzheimer’s Association; Alzheimer’s Drug Discovery Foundation; Araclon Biotech; BioClinica, Inc.; Biogen; Bristol-Myers Squibb Company; CereSpir, Inc.; Cogstate; Eisai Inc.; Elan Pharmaceuticals, Inc.; Eli Lilly and Company; EuroImmun; F. Hoffmann-La Roche Ltd and its affiliated company Genentech, Inc.; Fujirebio; GE Healthcare; IXICO Ltd.; Janssen Alzheimer Immunotherapy Research & Development, LLC.; Johnson & Johnson Pharmaceutical Research & Development LLC.; Lumosity; Lundbeck; Merck & Co., Inc.; Meso Scale Diagnostics, LLC.; NeuroRx Research; Neurotrack Technologies; Novartis Pharmaceuticals Corporation; Pfizer Inc.; Piramal Imaging; Servier; Takeda Pharmaceutical Company; and Transition Therapeutics. The Canadian Institutes of Health Research is providing funds to support ADNI clinical sites in Canada. Private sector contributions are facilitated by the Foundation for the National Institutes of Health (www.fnih.org). The grantee organization is the Northern California Institute for Research and Education, and the study is coordinated by the Alzheimer’s Therapeutic Research Institute at the University of Southern California. ADNI data are disseminated by the Laboratory for Neuro Imaging at the University of Southern California.

## Data Availability Statement

The original contributions presented in the study are included in the manuscript/supplementary material, further inquiries can be directed to the corresponding author/s.

## Ethics Statement

As per ADNI protocols, all procedures performed in studies involving human participants were in accordance with the ethical standards of the institutional and/or national research committee and with the 1964 Helsinki declaration and its later amendments or comparable ethical standards. The ADNI data collection was carried out after obtaining written informed consent from the participants. More details can be found at adni.loni.usc.edu.

## Author Contributions

TT and MD supervised and managed the research, and revised the manuscript. WaL leads the implementations, experiments, and wrote the manuscript. WeL co-lead the work and co-investigated the work. GC, HZ, and QG provided support to the work of experiments and revised the manuscript. YH gave support in data preprocessing. All authors contributed to the article and approved the submitted version.

## Conflict of Interest

QG and TT was partially employed by the company Imperial Vision Technology. The remaining authors declare that the research was conducted in the absence of any commercial or financial relationships that could be construed as a potential conflict of interest.
